# Vanishing Act: A Case Report of Missing Breast Tumour Marker

**DOI:** 10.7759/cureus.69737

**Published:** 2024-09-19

**Authors:** Shaleene Subramaniam, Anushya Vijayananthan, Kartini Rahmat

**Affiliations:** 1 Biomedical Imaging, University Malaya Medical Center, Kuala Lumpur, MYS; 2 Radiology, Hospital Sultan Ismail, Johor Bahru, MYS

**Keywords:** biopsy, mri, ultrasound, mammography, breast radiology, general surgery and breast cancer, breast cancer outcomes, general radiology, breast and endocrine surgery

## Abstract

Breast tissue markers are essential in localising tumours post-neoadjuvant chemotherapy prior to breast-conserving surgery. However, due to the advancement in neoadjuvant therapies, greater efficacy in reducing tumour size increases the possibility of marker migration, potentially compromising surgical outcomes. We report a case of a 34-year-old woman with left breast invasive carcinoma, where the tissue marker, placed under ultrasound guidance before chemotherapy, migrated and was undetectable after eight chemotherapy cycles. The delay in surgery was resolved by identifying the marker in the left pectoral muscle using CT, though proximity to the lung prevented hook wire placement. Proposed migration mechanisms include the "accordion effect" and haematoma-induced displacement, highlighting the dynamic nature of breast tissue. Various imaging modalities, such as mammography, ultrasound, and CT, have proven helpful for marker localisation. This case underscores the need for a deeper understanding of tissue dynamics and emphasises interdisciplinary communication to adapt treatment strategies. As medical knowledge continues to evolve, insights are needed to refine best practices in breast cancer management and radiological interventions.

## Introduction

Using breast tissue markers has demonstrated considerable utility and safety in localising tumours among patients undergoing neoadjuvant chemotherapy and breast-conserving surgery [1]. The migration of breast tissue markers is an established complication. Pronounced migration has the potential to impede the attainment of clear surgical margins, a concern that assumes heightened significance in the context of breast cancer due to the prevalent attainment of robust clinical responses following contemporary neoadjuvant systemic therapies. This renders markers a pivotal reference point for the residual lesion [2]. The migration mechanisms encompass the accordion effect, displacement facilitated by haematoma formation, and migration along the biopsy trajectory or within adipose breast tissue. This case presents an unusual occurrence involving the migration of a tissue marker in a 34-year-old female patient diagnosed with left breast invasive cancer. The tissue marker was found to have migrated onto the left pectoral muscle.

## Case presentation

**Figure 1:**
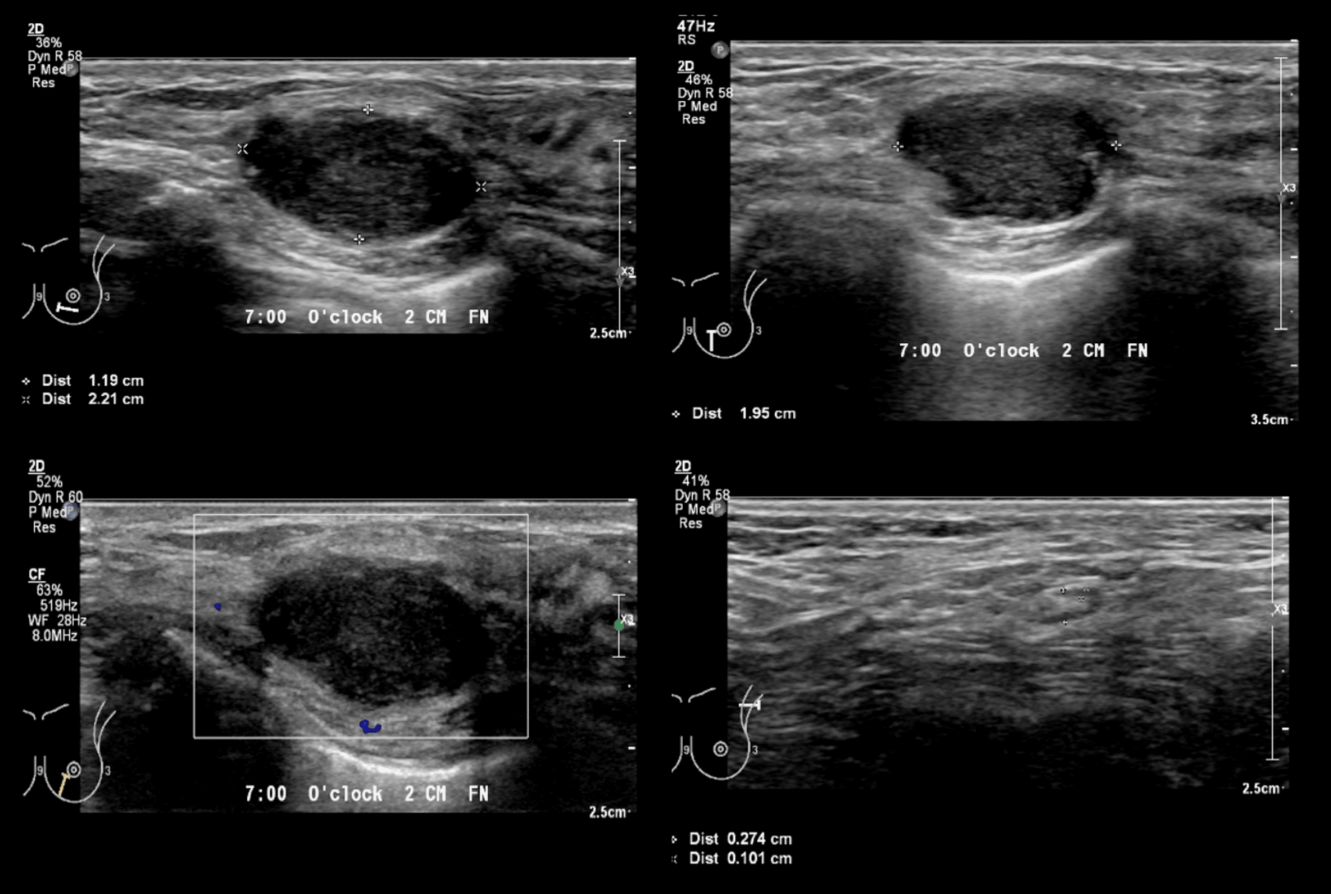
Left breast ultrasound showing micro-lobulated hypoechoic lesion at 7 o’clock 2 cm FN (1.2x2.2x2.0 cm). No internal vascularity, posterior features, or significant lymphadenopathy on the left. FN: From nipple

A 34-year-old BRCA1 gene carrier female patient was diagnosed with triple negative left breast invasive carcinoma (cT2 N0M0, stage 2a). She initially presented with a tender left breast lump. Screening ultrasound showed a suspicious left micro-lobulated hypoechoic lesion at 7 o’clock, 2 cm from the nipple, measuring 1.2x2.2x2.0 cm (Figure 1). No internal vascularity or posterior features were observed. Subsequently, an ultrasound core biopsy of the suspicious left breast lesion was performed, and the histopathological sample showed left breast invasive carcinoma, no specific type, modified Bloom and Richardson’s Grade 3.

**Figure 2:**
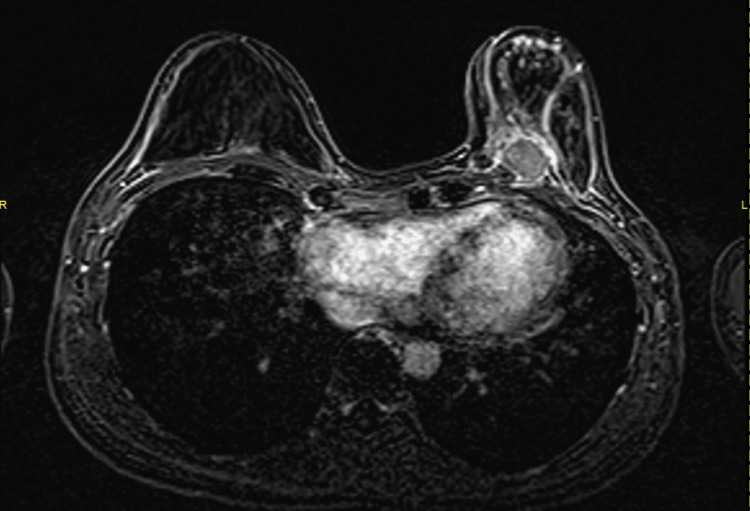
Dynamic contrast-enhanced MRI breasts in axial slice showing left 7 o'clock round rim-enhancing mass lesion with invasion of the underlying left pectoral muscle. No evidence of multifocality or multicentricity.

**Figure 3:**
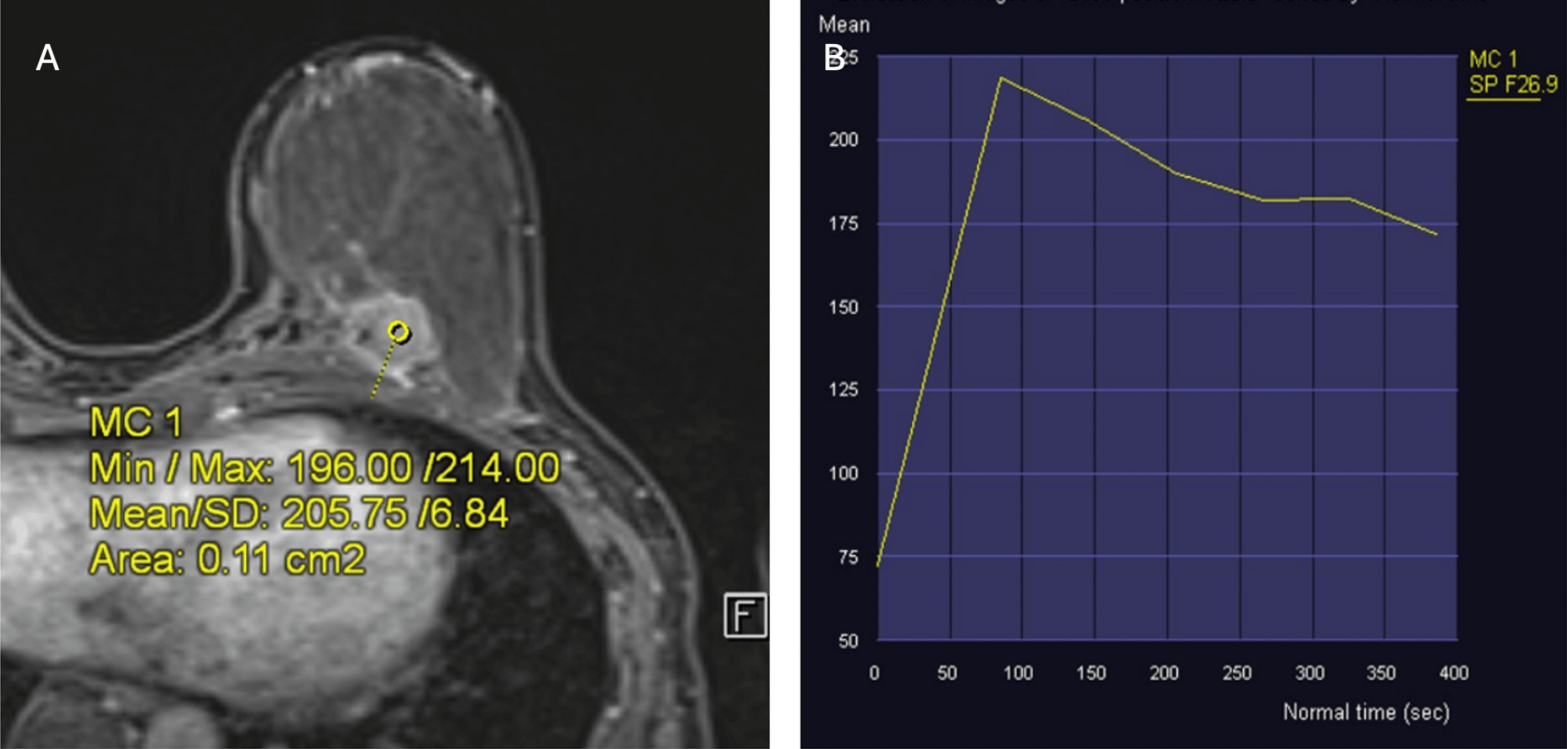
Dynamic contrast-enhanced MRI breast showing (A) placement of the ROI within the left breast 7 o'clock lesion rim-enhancing lesion, and (B) Type 3 kinetic curve produced for the corresponding lesion. ROI: Region of interest

The patient underwent an MRI breast assessment, which showed a round circumscribed rim-enhancing mass lesion with the invasion of the underlying pectoral muscle at 7 o’clock, 7.2 cm from the nipple (Figure 2). This lesion demonstrated a Type 3 kinetic curve with rapid contrast enhancement with washout in the delayed phase (Figure 3). Staging CT showed no evidence of distant metastasis.

**Figure 4:**
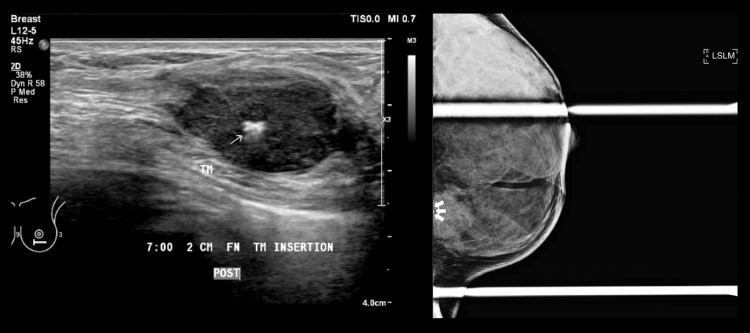
Tumour marker insertion was done under ultrasound guidance. The placement of the marker was confirmed using the SLM view on the mammogram (white arrows). SLM: Straight mediolateral

Due to the size and aggressive nature of the tumour, the treatment plan made for the patient during the multidisciplinary team meeting was for neoadjuvant chemotherapy to shrink the tumour before surgery and after a complete pathological response, the surgeons were to proceed with breast-conserving surgery. Prior to the commencement of chemotherapy, a tissue marker was inserted into the left breast lesion under ultrasound guidance. The placement of the tissue marker was further confirmed using a post-procedural mammogram (Figure 4).

**Figure 5:**
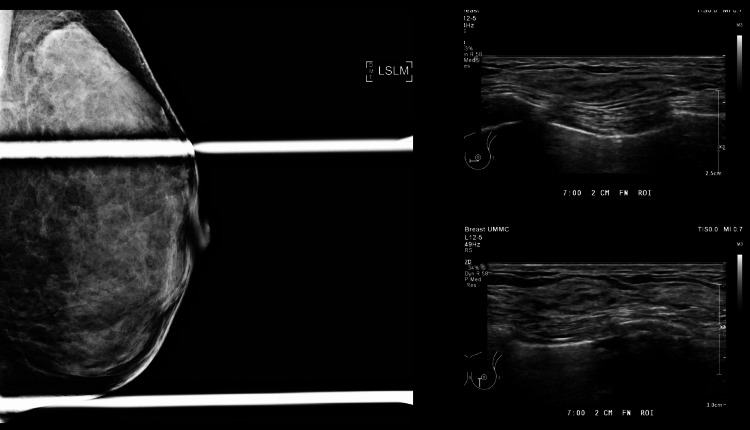
Post eight cycles of chemotherapy reassessment study showing non-visualisation of the left breast primary lesion and tumour marker on both ultrasound and mammogram.

After eight cycles of neoadjuvant chemotherapy, initially doxorubicin + dyclophosphamide and later a paclitaxel + carboplatin regime, she was reassessed using a mammogram and ultrasound. During this study, the left breast 7 o’clock lesion was not visualised. The tissue marker, however, was also not seen despite best efforts (Figure 5).

**Figure 6:**
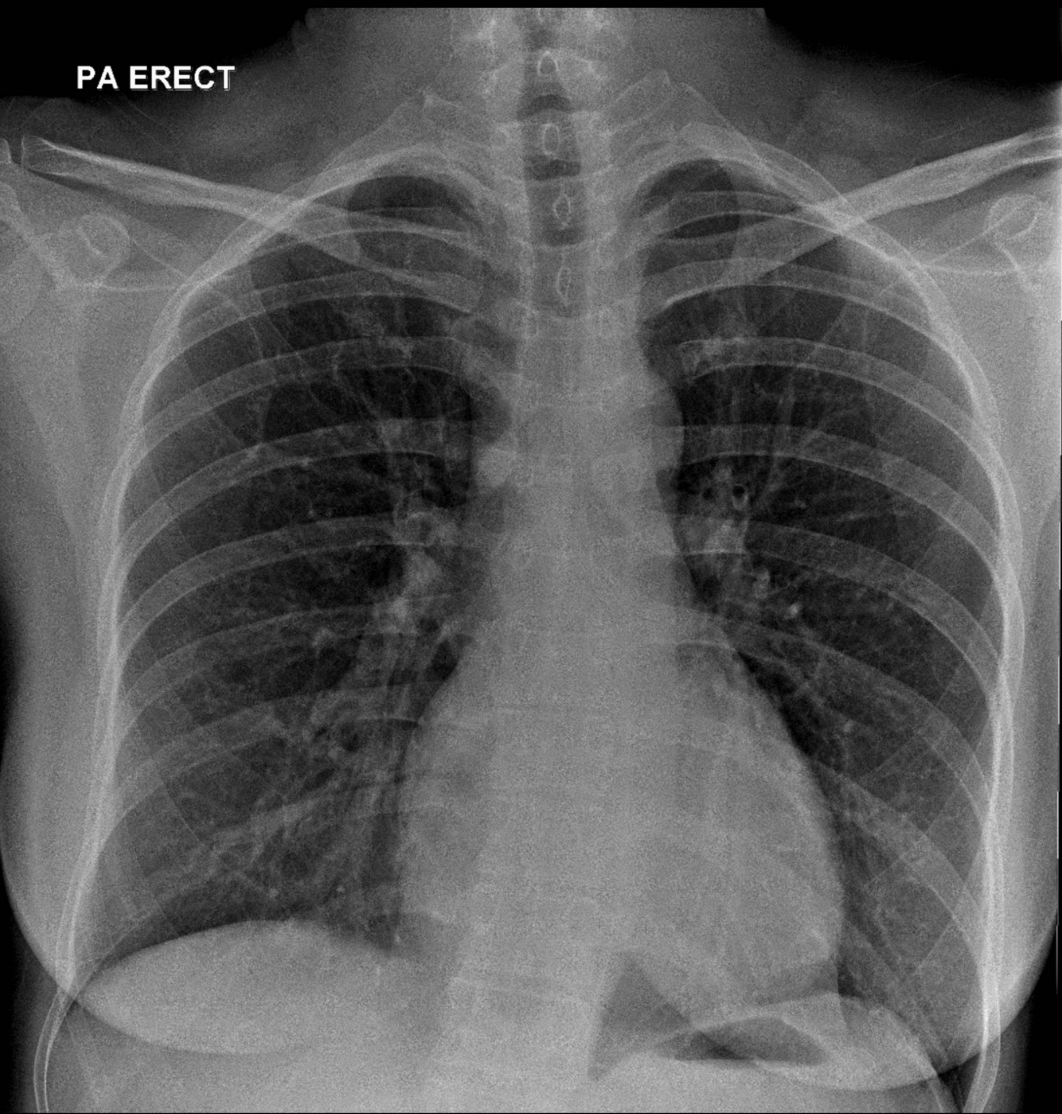
Supplementary chest radiograph did not show radioopacity at the ROI. ROI: Region of interest

**Figure 7:**
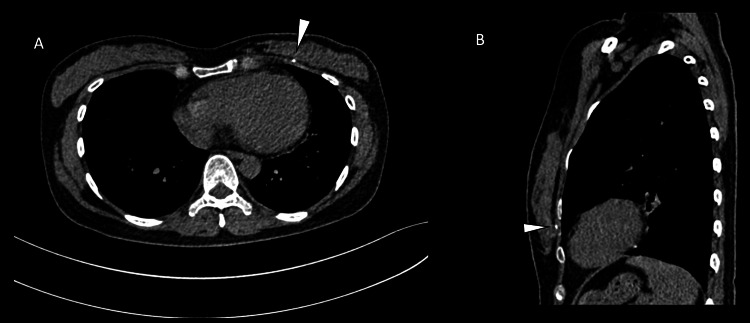
CT-guided fluoroscopy in axial (A), sagittal (B) shows a dense foreign object (white arrowheads) lying posterior to the left pectoral muscle. Due to its compromising position, the decision to insert a hook wire was not taken.

**Figure 8:**
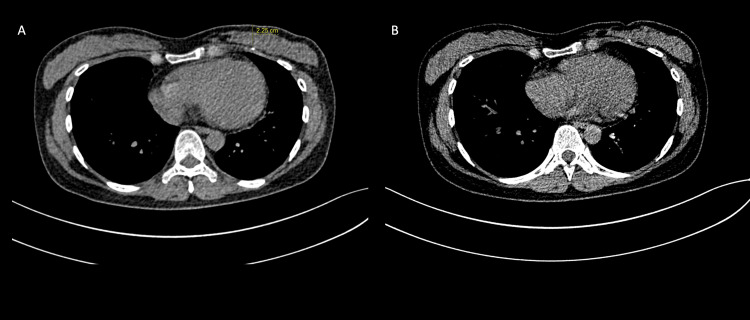
CT-guided fluoroscopy revealing the tumour marker positioned at a depth of 2.2 cm from the skin (A). An 'X' demarcation was meticulously placed on the cutaneous surface directly above the tumour marker, facilitating precise localisation for our surgical colleagues.

A supplementary chest radiograph was done, but we could still not identify the tissue marker (Figure 6). The patient was then planned for hook wire localisation of the tissue marker under CT guidance. A dense foreign body was seen posterior to the left pectoral muscle during the preliminary scan (Figure 7). The hook wire was not inserted as the chances of injuring the lung parenchyma were very high. An ‘X’ was marked on the skin, overlying the tissue marker to aid the surgeons (Figure 8). She was then brought to the operating room, where a left breast-conserving surgery, sentinel lymph node biopsy, and anterior intercostal artery perforator (AICAP) flap transposition were performed. The frozen section was not sent as the surgeons could not identify the tissue marker intra-operatively. Histopathological examination results of the sample obtained showed no residual invasive carcinoma and clear margins with a complete pathological response. The patient subsequently underwent adjuvant three-dimensional conformal radiotherapy (3DCRT) with a sequential boost to the tumour bed to consolidate the therapeutic outcomes.

## Discussion

This case involves a patient with invasive breast carcinoma, wherein an Ultraclip titanium breast tissue marker migrated prior to planned surgery, prompting a deeper understanding of the mechanisms underlying such displacement. Initially positioned within the lesion to guide surgical resection post-neoadjuvant chemotherapy, the marker was unexpectedly found adjacent to the left pectoral muscle, necessitating a reassessment of the surgical strategy.

The Ultraclip titanium breast tissue marker was chosen because of its properties that minimise migration risk [2,3]. Constructed from biocompatible, inert titanium, the marker resists chemical reactions and adverse tissue responses. Its mechanical integrity and potential anchoring features help maintain its position despite the mechanical stresses of breast tissue mobility [4]. The marker's radiopaque visibility aids in accurate tracking during imaging, which is crucial for treatment planning [5].

Various radiological methods can be employed to address the challenge of locating the displaced Ultraclip titanium marker. One practical approach involves obtaining additional cleavage views during mammography [6]. The cleavage view is specifically designed to optimise visualisation of the breast tissue in the central region, where the marker's migration likely occurred. By capturing multiple angles in this area, the chances of identifying the migrated marker and assessing its proximity to surrounding structures are significantly enhanced.

Other methods include ultrasound and colour Doppler imaging, which distinguishes the marker's appearance from surrounding tissues [1]. Contrast-enhanced MRI improves marker detection by employing contrast agents to enhance structures' visibility. The Ultraclip marker's radiopacity could be accentuated by surrounding tissue enhancement, making identifying against the background tissue easier. Notably, in this case, a CT-guided fluoroscopic procedure was employed for marker identification and labeling prior to surgical excision, underlining the utility of interventional radiology approaches.

The migration mechanisms proposed to explain the displacement of the tissue marker encompass the "accordion effect," displacement facilitated by haematoma formation, and migration along the biopsy trajectory or within the adipose breast tissue [3,7]. Each of these mechanisms underscores the dynamic and intricate nature of breast tissue, which can contribute to the unintended movement of foreign bodies within the breast.

The "accordion effect" refers to the notion that markers placed within the breast tissue can experience displacement due to the inherent mobility and pliability of the breast tissue itself. During routine movements or due to changes in breast shape, the marker might shift from its original location, potentially leading to its migration over time. This emphasises the need for a comprehensive understanding of breast tissue behavior and its potential impact on market stability.

Haematoma formation as a potential facilitator of marker migration underscores the significance of post-biopsy changes within the breast tissue. Haematomas can create localised pressure gradients, forcing the marker to move away from its original position. The interaction between haematoma formation and the marker's physical properties, such as size and weight, can influence the direction and extent of migration.

Migration along the biopsy trajectory or within adipose breast tissue highlights the intricate pathways that markers can follow within the breast. The presence of biopsy tracts and the variable composition of breast tissue can lead to unexpected routes of displacement. This phenomenon serves as a reminder that the anatomical complexities of the breast demand careful consideration when planning radiological procedures and subsequent interventions.

The case report also underscores the importance of meticulous communication and collaboration among radiologists, oncologists, and surgeons. The unexpected migration of the tissue marker altered the surgical approach and necessitated a reassessment of the tumour's localisation. Such situations require interdisciplinary efforts to adapt the treatment plan effectively, ensuring the patient receives the best care.

## Conclusions

In conclusion, the case report of a missing tissue marker in a patient with invasive breast carcinoma highlights the intricacies between breast tissue dynamics and radiological interventions. The proposed migration mechanisms-accordion effect, haematoma-facilitated displacement, and migration along biopsy trajectory-underscore the need for a comprehensive understanding of breast anatomy and behaviour. This case also emphasises the importance of effective interdisciplinary communication and adaptation of treatment strategies in response to unforeseen challenges. As medical knowledge continues to evolve, insights from such cases contribute to refining best practices in breast cancer management and radiological interventions.
